# 

**Published:** 1902-09

**Authors:** 


					﻿The Fourteenth International Congress of Medicine will be held
in Madrid, Spain, from April 23d to 30th, 1903, under the patron-
age of their Majesties, the King of Spain and the Queen-Mother.
The President of the Congress is Professor Julian Callejay San-
chez, the General Secretary is Dr. Angel Fernande-Caro, and the
General Treasurer is Professor Jose Gomez Ocana. The prelim-
inary statement of regulations and program has just been issued,
and it announces that the members of the Congress will be physi-
cians, pharmacists, dentists, veterinary surgeons, and other persons
working at branches of medical science, both Spaniards and for-
eigners, who have entered their names and paid their subscriptions.
Other persons who possess scientific or professional titles, and who
wish to take part in the work of the Congress may share in it un-
der the above conditions. The subscription is thirty pesetas, and
this sum must be paid before the opening of the Congress to the
General Secretary, Faculty of Medicine, Madrid. A card of mem-
bership will sent to the subscriber. Until March 20th, 1903, sub-
scriptions may be paid to the Secretary of the National Committee
of the subscriber, but after that date subscriptions must be paid
directly to the General Secretary at Madrid. Members will re-
ceive a summary of the proceedings of the Congress, and a full
report of the particular section which they join. The official lan-
guages of the Congress will be Spanish, French, English, German
and Italian. Papers must be sent to the General Secretary be-
fore January 1st, 1903, to be certain of a place in the order of
business. Papers presented later will be considered after the dis-
cussion of those regularly announced. Communications should be
accompanied by a short abstract, which will be printed and distrib-
uted among the members of the Congress. J. II. Huddleston,
Secretary American Committee, 126 West 85th St., New York
City.
This Journal and Atlanta Semi-weekly Journal one year for $1.00
cash in advance. No checks. Strictly to new subscribers.
NOTICE.— This Journal and the International Journal
of Surgery one year for $ 1.00 cash, to new subscribers. No
out-of-town checks accepted unless 10c. is added for cost of
cashing. Strictly to new subscribers.
NOTICE.—This JOURNAL and THE INTERNATION-
AL JOURNAL OF SURGERY one year for $1.00 cash,
to new subscribers. No out-of-town Checks ac-
cepted unless lOc. is added for cost of cashing.
This Journal and Atlanta Semi-weekly Journal one year for $1.00
cash in advance. No checks. Strictly to new subscribers.
				

